# “How Long Have I Got?” in Stage IV NSCLC Patients With at Least 3 Months Up to 10 Years Survival, Accuracy of Long-, Intermediate-, and Short-Term Survival Prediction Is Not Good Enough to Answer This Question

**DOI:** 10.3389/fonc.2021.761042

**Published:** 2021-12-21

**Authors:** Huiru Guo, Hegen Li, Lihua Zhu, Jiali Feng, Xiange Huang, Jan P. A. Baak

**Affiliations:** ^1^ Department of Medical Oncology, Longhua University Hospital, Shanghai, China; ^2^ Department of Pathology, Stavanger University Hospital, Stavanger, Norway; ^3^ Medical Practice Dr. Med Jan Baak AS, Tananger, Norway

**Keywords:** nonsmall cell lung cancer, stage IV, outcome prediction, long-term survival, baseline features, treatment factors

## Abstract

**Background:**

Most lung cancer patients worldwide [stage IV nonsmall cell lung cancer (NSCLC)] have a poor survival: 25%–30% die <3 months. Yet, of those surviving >3 months, 10%–15% (70,000–105,000 new patients worldwide per year) survive (very) long. Surprisingly, little scientific attention has been paid to the question, which factors cause the good prognosis in these NSCLC stage IV long survivors. Therefore, “How long do I still have?” currently cannot be accurately answered. We evaluated in a large group of 737 stage IV NSCLC patients surviving 3.2–120.0 months, the accuracies of short- and long-term survival predictive values of baseline factors, radiotherapy (RT), platinum-based chemotherapy (PBT), and tyrosine kinase inhibitor targeted therapy (TKI-TT).

**Methods:**

This is a noninterventional study of 998 consecutive first-onset stage IV NSCLC patients. A total of 737 (74%) survived 3.2–120.0 months, 47 refused RT, PBT, and TKI-TT. Single and multivariate survival analysis and receiver operating curve (ROC) analysis were used with dead of disease (DOD) or alive with disease (AWD) as endpoints.

**Results:**

The median survival (16.1 months) of 47 patients who refused PBT, RT, and TKI-TT was significantly worse than those with RT, PBT, and/or TKI-TT (23.3 months, HR = 1.60, 95% CI = 1.06–2.42, *p* = 0.04). Of these latter 690 patients, 42% were females, 58% males, median age 63 years (range 27–85), 1-, 2-, 5-, and 10-year survival rates were 74%, 49%, 16%, and 5%. In total, 16% were alive with disease (AWD) at the last follow-up. Pathology subtype (adenocarcinoma vs. all others), performance score, TNM substage, the number of PBT cycles and TKI-TT had independent predictive value. However, with the multivariate combination of these features, identification results of short-term nonsurvivors and long-term survivors were poor.

**Conclusions:**

In stage IV NSCLC patients with >3 months survival, baseline features, and systemic therapeutic modalities have strong survival predictive value but do not accurately identify short- and long-term survivors. The predictive value of other features and interventions discussed should be investigated in the worldwide very large group of stage IV NSCLC patients with >3 months survival.

## Introduction

Lung cancer is the leading cause of cancer-related mortality, and the global overall 5-year survival rate of nonsmall cell lung cancer (NSCLC) is less than 20% (1). Similar rates are found in the People’s Republic of China (2). With 2 million new lung cancers worldwide in 2020 (3), about 1.6 million are NSCLC, and of these, 60%–70% are in advanced stage IV at the time of diagnosis. Thus, the annual worldwide number of new NSCLC stage IV patients is close to 1 million.

Patients with metastatic (stages IIIB–IV) NSCLC have such a poor life expectancy that surgery in general is not recommended, leaving platinum-based therapy (PBT), radiotherapy (RT), tyrosine kinase inhibitor target therapy (TKI-TT), or immunotherapy as the treatment options. The response rate of the standard first-line chemotherapy of stages IIIB–IV lung cancers has improved significantly from 4–6 to 8–10 months; 1-year survival rate is ≥30% (4). Moreover, tyrosine kinase inhibitors (TKI) (5) can significantly prolong survival, especially in epidermal growth factor receptor (EGFR) mutation-positive NSCLCs. In Asian patients, EGFR-mutation status was 51% positive (6), but in Western countries, such as Norway, it is much lower (8%) (7).

Of the metastatic NSCLC patients, those with stage IV (8, 9, current patient material) has an especially poor prognosis as 25%–30% are dead of disease (DOD) <3 months after initial diagnosis despite RT, PBT, and/or TKI-TT. As far as we know, large, long-term studies in this stage IV NSCLC with at least 3 months survival are lacking. In the patients to be described in the current article, 10%–15% survived between 60 and 75 months. At 10 years follow-up, still 5% was alive with distant metastases. This latter percentage may seem low, but speaks much more, when it is considered that with worldwide 700,000 new NSCLC stage IV patients who annually survive >3 months, even 5% regards yearly 35,000 new stage IV alive-with-disease (AWD) patients with 10 years follow-up.

The decision for systemic therapy of stage IV NSCLC patients usually depends on performance score (PS) 0–1, adenocarcinoma, presence of EGFR mutation, and age 18–75.

However, surprisingly, truly little scientific attention has been paid to the factors causing the good prognosis in the worldwide 35,000–105,000 stage IV NSCLC long survivors. As scientific support currently is lacking, the question from individual stage IV NSCLC patients surviving at least 3 months: “How long do I still have?,” currently cannot be accurately answered.

We evaluated in a large group of 737 stage IV NSCLC patients surviving 3.2–120.0 months whether baseline patient-, tumor-, and treatment factors can accurately predict long- and short-term survival.

## Materials and Methods

### Ethics and Patients

This noninterventional, retrospective observational study on patients with first-time onset stage IV NSCLC was approved by the Institutional Research Board of the Longhua University Hospital (LUH), Xuhui district, Shanghai, China before the study commenced. JB later got permission from the Research Director of the Stavanger University Hospital, Stavanger, Norway, to participate. The study was performed in accordance with the Declaration of Helsinki (2013) for experiments involving humans and in line with the recommendations for the conduct, reporting, editing, and publication of scholarly work in medical journals. The work complies with the principles laid down in the CIOMS International Ethical Guidelines for Biomedical Research Involving Human Subjects (2002). All patients have approved and signed an agreement before they received treatment, to use their data in noninterventional studies without any changes in their treatment. These signed papers are saved in the patient record of each patient. As this is an observational noninterventional study, it did not add any risk for the patients.

All consecutive patients diagnosed at the LUH between January 1, 2009–December 31, 2018 were considered. There were originally 998 pathologically confirmed first onset stage IV NSCLC, of which 737 survived 3.2–120.0 months.

The data were retrieved from the LUH patients’ records, by four well-trained, experienced medical oncologists. Each item in every patient record was carefully evaluated and registered. For quality control, in a random 10% of the records, the items were controlled by another independent medical oncologist and consensus was always obtained.

Dead of disease (DOD) or alive with disease (AWD) were used as endpoints. There were originally 998 pathologically confirmed first-onset stage IV NSCLC, 737 survived 3.2–120.0 months. Patients were treated with/without RT and/or PBT and/or TKI-TT.

Stage was defined according to the Eighth Edition of the TNM Classification for Lung Cancer as IVA or IVB (8).

The Eastern Cooperative Oncology Group PS was used, defined as follows: 0 = Fully active, able to carry on all pre-disease performance without restriction; 1 = Restricted in physically strenuous activity but ambulatory and able to carry out work of a light or sedentary nature, e.g., light house work, office work; 2 = Ambulatory and capable of all selfcare but unable to carry out any work activities; up and about more than 50% of waking hours; 3 = Capable of only limited selfcare; confined to bed or chair more than 50% of waking hours; 4 = Completely disabled; cannot carry on any selfcare; totally confined to bed or chair; 5 = Dead. As in previous studies, the PS was dichotomized as 0–1 vs. ≥2, as the latter patients have a much worse outcome expectation.

The smoking habit index was defined by the number of cigarettes smoked per day (as indicated by the patient) times the number of years smoked (e.g., with 20 cigarettes per day, for 25 years, results in a Smoking Index = SI is 500). As many patients were heavy smokers, we used ≤ 500 vs. > 500 as the thresholds to distinguish between “Nonsmoker plus Light-smokers” and “Heavy smokers”.

### Details of Chemotherapy and Pharmaceutical Intervention for Nausea and Vomiting, Radiotherapy, and Targeted Therapy

The patients were randomly treated with one of the following regimens (there were no differences in the outcome of lung cancer with different platinum-based regimens (4) (1): NP: vinorelbine 25 mg/m^2^ was administered on days 1 and 8, and cisplatin 75 mg/m^2^ was administered on day 1 of a 4-week cycle (2). TP: paclitaxel 135 mg/m^2^ and cisplatin 75 mg/m^2^ were administered on day 1. The cycle was repeated every 4 weeks (3). GP: gemcitabine 1,250 mg/m^2^ was administered on days 1 and 8, and cisplatin 75 mg/m^2^ was administered on day 1 of a 4-week cycle. The standard of care for initial treatment of advanced NSCLC has been four to six cycles of platinum-based chemotherapy followed by close observation. This approach was based on studies that suggested increased toxicity with no improved clinical benefit when the platinum doublet was continued until disease progression (9). Patients with grades 1–3 nausea or vomiting routinely received pharmaceutical antiemetic ondansetron (Zofran) treatment.

RT was given for palliative reasons. The dosages for body tumors were 50–55 Gray/25–30 times, and for brain tumors, total dosage was 30 Gray/10–15 times. For stage IV NSCLC, radical palliative thoracic RT with a median dose of 55 Gray was given, as this is safe and might be beneficial for primary lung lesions of metastatic NSCLC patients with controlled extrathoracic diseases (10).

The following first-line TKIs for treating advanced lung cancer with epidermal growth factor receptor (EGFR) (11–14) mutation (+) were used: gefitinib (Iressa^®^), erlotinib (Tarceva^®^), and icotinib (Conmana^®^). The latter is a highly selective, first-generation epidermal growth factor receptor tyrosine kinase inhibitor (EGFR-TKI) for use as first-line monotherapy in patients with NSCLC with somatic EGFR mutations. Currently, it is solely approved and marketed in China. When the first-line TKIs failed, any of the following TKIs was given: osimertinib (Tagrisso^®^), afatinib (Gilotrif^®^).

### Statistical Methods

SPSS version 25 (IBM-SPSS, Armunk, New York, USA) and MedCalc version 20.014 (MedCalc Software Ltd, Ostend, Belgium) were used for statistical analyses. Descriptive statistics were assessed for all features. For survival analyses, continuous features were discretized according to medians, tertiles, quartiles, or the results of receiver operating curve (ROC) analysis. DOD and AWD at the last follow-up were used as endpoints. Clinical impressions suggested that patients ≥75 years more often have a worse survival; this was therefore especially analyzed. Single and multivariate survival (Cox model) and binary logistic regression analysis were used. To evaluate the influence of the duration of the follow-up, as a cofounder in the regression analysis, the quartiles of follow-up duration were used as a covariate.

## Results

### Univariate Results

Of the total group of 737 stage IV NSCLC patients, 47 refused chemotherapy, radiotherapy, and TKI-TT. Their median survival was 16.1 months, significantly worse than those who received either chemotherapy, TKI-TT, or both (HR = 1.60, 95% CI = 1.06–2.42, *p* = 0.04). Their median age was 72 (range 41–85), i.e., higher than that of the other 690 patients (which were median 63 years, *p* = 0.0009). In 28%, their performance scores were >1 (compared with 17% in the other 690 patients, *p* = 0.07). On the other hand, TNM IVA and IVB were 30% and 70%, which was not different from the other 690 patients (*p* = 0.39). Thus, they were older, had a (nonsignificant) trend towards higher PS, but TNM stages were not different from the other 690 patients. We therefore assume, that not only their slightly higher age and PS but also fear for side effects of the treatments may have been the major factors for them to refuse treatment.

Of the other 690 patients, 288 were females (42%) and 402 males (58%). Median age was 63 years (range 27–85), median survival was 23.3 months (range 3.2–120.0), and 1-, 2-, 5-, and 10-year survival rates were 74%, 49%, 16%, and 5%; 112/690 = 16% of the patients were AWD at the last follow-up. Median survivals for the dead-DOD and AWD patients were 20.0 and 35.0 months. **Table 1** shows the distribution of the different features and their univariate prognostic value.

**Table 1:** Univariate survival analysis in 690 stage IV NSCLC patients with at least 3 months survival after primary diagnosis, using AWD or DOD as endpoints.

Characteristic	Dead of disease/at risk	% Censored (alive with disease)	Median survival time (months)	Probability of no difference^a^	Hazard ratio^a^	95% confidence interval^a^
**Baseline features**						
**Total**	578/690	16%	23.3			
**Age (years)**						
***<57***	168/191	12%	27.0			
***57–63***	138/171	19%	23.0		1.00	0.81–1.26
***64–69***	142/181	22%	24.0		1.00	0.80–1.25
***≥70***	130/147	12%	21.0	0.59	1.14	0.91–1.45
***<75***	497/601	17%	25.0			
***≥75***	81/89	9%	17.8	0.006	1.46	1.11–1.91
**Gender**						
***Females***	233/288	19%	31.0			
***Males***	345/402	14%	21.0	0.0002	1.37	1.16–1.61
**Smoking habit index**						
***No plus light***	406/488	17%	26.0			
***Heavy***	172/202	15%	20.0	0.003	1.33	1.10–1.62
**Performance score**						
***0–1***	463/571	19%	26.0			
***>1***	115/119	3%	17.0	<0.0001	1.91	1.49–2.45
**TNM**						
***IVA***	197/248	21%	31.2			
***IVB***	381/442	14%	19.0	<0.0001	1.39	1.18–1.64
**Pathology**						
***Adenocarcinomas***	398/490	19%	27.4			
***Squamous cell***	77/88	13%	18.0		1.43	1.10–1.88
***Other NSCLC subtypes***	103/112	8%	17.1	<0.0001	1.59	1.24–2.04
**Sum of TNM, performance score, and pathology subtypes**						
***Sum = 3 (TNM = 4A) + (PS = 0–1) + pathology = adenocarcinoma)***	109/151	28%	39.0			
***Sum = 4 (different other combinations of TNM, PS, and pathology)***	286/342	16%	23.0		1.47	1.22–1.78
***Sum = 5 (different other combinations of TNM, PS, and pathology)***	159/172	8%	17.0		2.25	1.77–2.87
***Sum = 6 (TNM = 4B) + (PS = 2–4) + pathology = no adenocarcinoma)***	24/25	4%	10.0	<0.0001	3.28	1.79–6.03
**EGFR mutations^b^**						
***No***	0/152	14%	23.0			
***Mut-19***	16/26	38%	28.0			
***Mut-20***	1/2	50%	14.0		0.72	0.47–1.10
***Mut-21***	9/20	55%	44.1		0.47	0.30–0.73
***Others***	14/18	22%	23.3	0.04	0.98	0.57–1.66
**Therapeutic modalities**						
**Radiotherapy**						
***No***	378/462	18%	22.5			
***Yes***	200/228	12%	25.3	0.69	0.97	0.81–1.15
**Number of chemocycles**						
***0***	45/60	25%	28.0			
***2***	1/1	0%	3.2	–	–	–
***3***	14/14	0%	4.0	–	–	–
***4–6***	518/615	16%	24.0	<0.0001	–	–
**Targeted therapy**						
***No***	422/494	15%	22.0			
***Tarceva***	36/40	10%	17.7		0.97	0.69–1.38
***Iressa***	99/127	22%	34.0		0.74	0.61–0.91
***Conmana***	16/24	33%	50.0		0.57	0.39–0.85
***Second-line TKIs^c^***	5/5	0%	9.0	0.007	1.81	0.53–6.20

NSCLC, nonsmall cell lung cancer; PS, Eastern cooperative oncology group performance score; EGFR, epidermal growth factor receptor; Mut, mutation; TKI, tyrosine kinase inhibitor.

a Minus sign (−) means cannot be calculated, divided by zero error.

TNM, PS, and pathology were coded as follows: TNM 4A = 1, TNM4B = 2, PS = 0–1 = 1, PS >1 = 2; pathology = adenocarcinoma = 1; all other pathology subtypes = 2. The total sum could thus be 3 [(TNM = 4A=1) + (PS = 0–1 = 1) + pathology = adenocarcinoma = 1)], 6 [(TNM = 4B =2) + (PS >1 = 2) + pathology = squamous or other cell types = 2)], 4 (one of the three features = 2, the other two = 1) or 5 (2 of the 3 features were 2, the other one was 1).

b Based on determinations in 218 of the 690 patients.

c Osimertinib, afatinib.

Of the patients receiving TKI-TT, 58 of 60 did not get chemotherapy. Note the good survival rate of this no-chemotherapy subgroup.

Several of the TKI-TT (both Iressa and Tarceva) patients received the treatment without EGFR-mutation analysis. Many of these had squamous or large cell pathology subtypes and had the same poor survival as non-TKI-TT patients. These patients should never have received TKI-TT. This explains the somewhat less favorable outcome in the TKI-TT patients than described before in patients with EGFR mutations (5).

The following features had univariable predictive AWD vs. DOD survival value. Age (<75 vs. > 74 years, *p* = 0.006), gender (female vs. male, *p* = 0.0002), smoking habit index (no-plus-light vs. heavy smokers, *p* = 0.003), performance score = PS (≤1 vs. ≥ 2, *p* < 0.0001), TNM substage (IVA vs. IVB, *p* < 0.0001), pathological type (adenocarcinomas vs. all other NSCLC subtypes, *p* < 0.0001), sum of TNM + PS + pathology (*p* < 0.0001), EGFR mutations (no vs. yes, *p* = 0.04), the number of platinum-based-chemotherapy (PBT) cycles (*p* < 0.0001), and TKI-TT (*p* = 0.007).

### Multivariate Analysis Results

**Figure 1:**
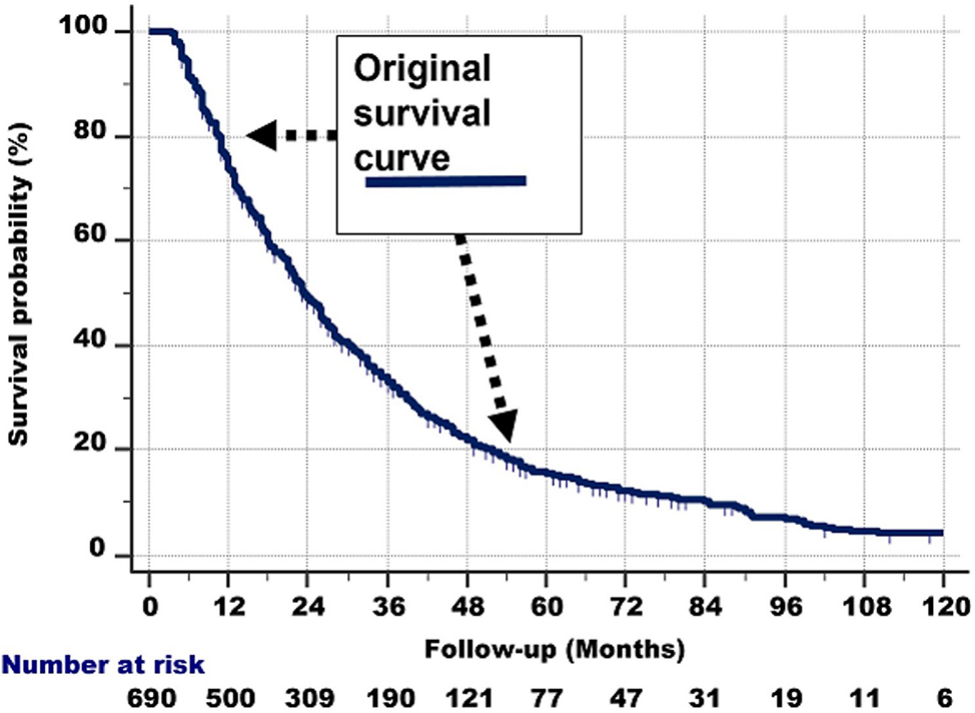
The survival curve of the 690 patients. As it is curved (i.e., not linear), it is probable that the total group consists of subgroups with different survival rates.

The shape of the survival graph of the 690 patients (**Figure 1**) is curved. This suggests that the group is heterogeneous, i.e., consists of subgroups with different survival rates.

We therefore studied by multivariate Cox regression analysis (both with Enter and Stepwise models), if short- and long-term survivors could be identified with the patient, tumor, and treatment characteristics. First, all features and then the univariably significant ones were included. These 2 different approaches selected the same features (**Table 2**).

**Table 2:** Multivariate survival analysis results in 690 stage IV nonsmall cell lung cancer patients with at least 3 months survival after primary diagnosis, using dead of disease and alive with disease at the last follow-up as endpoints (Chi-square 92.9, *P* < 0.0001).

Covariate	Beta	Standard error	Wald	Probability of no difference	Exp Beta	95% confidence interval of Exp Beta
**Age: *1 = <75, 2 = ≥75***	0.18	0.123	2.07	0.15	1.19	0.95–1.52
**Gender: *1 = female, 2 = male***	0.18	0.097	3.35	0.07	1.19	0.98–1.44
**Smoking habit index: *1 = no-plus-light, 2 = heavy***	0.07	0.104	0.50	0.48	1.08	0.88–1.32
**Pathology: *1 = adenocarcinoma, 2 = All others***	0.29	0.098	8.79	***0.003***	1.34	1.10–1.62
**Performance score: *1 = ＜2, 2 = ≥2***	0.48	0.107	20.45	***<0.0001***	1.62	1.31–2.00
**TNM: *1 = stage IVA, 2 = stage IVB***	0.34	0.091	14.00	***0.0002***	1.41	1.18–1.68
***Radiotherapy 0 = No, 1 = Yes***	−0.11	0.090	1.48	0.22	0.89	0.75–1.07
**Chemotherapy cycles: *1 = <4, 2 = 4–6***	−0.92	0.152	36.60	***<0.0001***	0.40	0.30–0.54
**TKI-targeted therapy** ***0 = No, 1 = Yes***	−0.10	0.049	4.27	***0.04***	0.90	0.82-0.99

TKI, tyrosine kinase inhibitor. Bold values is: “P < 0.05”.

The following features had independent prognostic value: pathology (*p* = 0.003), performance score (*p* < 0.0001), TNM substages (*p* = 0.0002), the number of chemotherapy cycles (*p* < 0.0001), and TKI-TT (*p* = 0.04) to predict survival (Chi-square = 92.9, *p* < 0.0001). None of the other features (age, gender, smoking habits index, radiotherapy) had additional value.

The same procedure was then repeated, but now for the 615 patients who had received the full 4–6 chemocycles (excluding all patients with 0, 2, and 3 chemocycles). The results were comparable. We concluded that the results are robust.

Moreover, including the quartiles of follow-up duration as a confounding covariate in the regression analysis showed that the duration of the follow-up did not influence these results.

**Figure 2:**
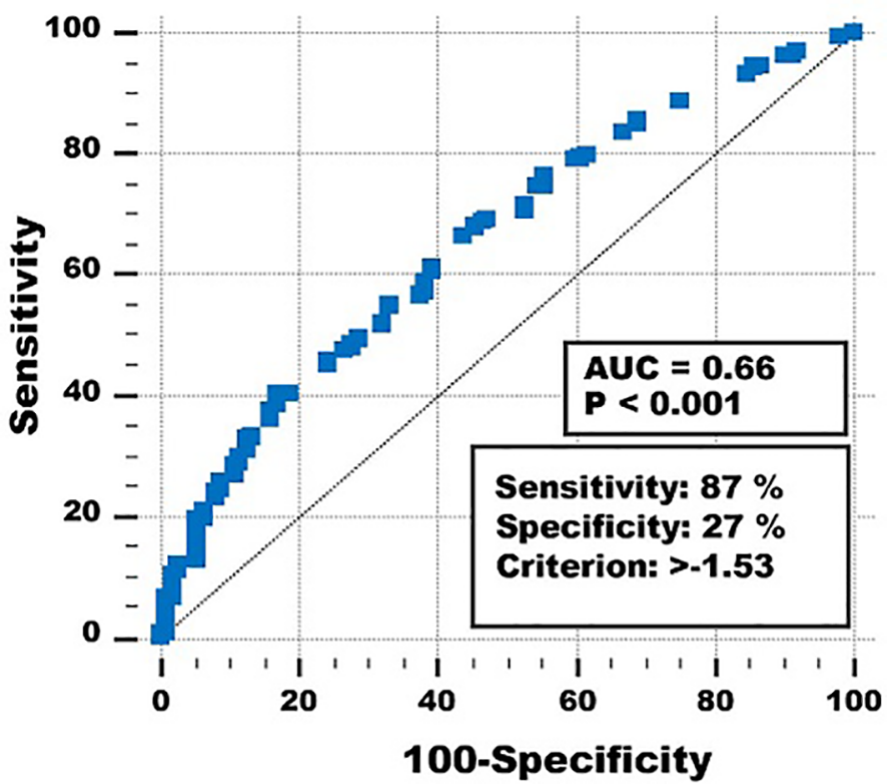
Receiver operating curve of the multivariate classifier to predict short- and long-term survival.

ROC analysis (area under the curve 0.66, *p* < 0.001) showed that the optimal prediction threshold for the multivariate classifier with the abovementioned variables and coefficients was < −1.53 vs. ≥ −1.53 (**Figure 2**).

Using this threshold for the multivariate prognostic classifier resulted in sensitivity = 87%, specificity = 27%, overall correctly classified cases 73%, and positive and negative predictive values 80% and 38%. Different thresholds gave higher specificities and negative predictive values, but at the expense of sensitivities, overall correctly classified cases and positive predictive values.

### Prediction of Short- and Long-Term Survival

**Figure 3:**
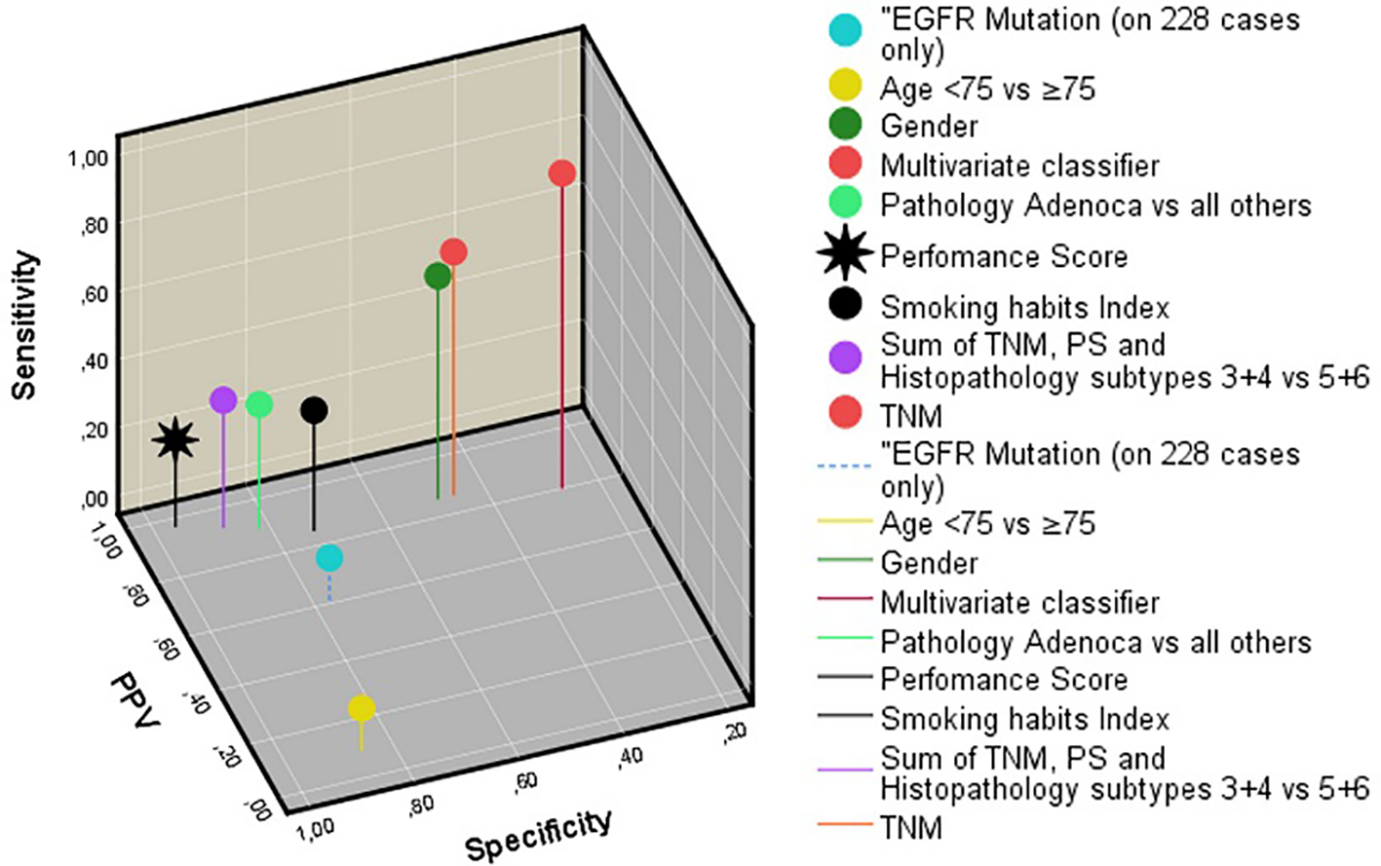
Three-dimensional representation of the sensitivity, specificity, and positive predictive value of all features studied. Note that the multivariate classifier gives the best results. EGFR, epidermal growth factor receptor. PS, Eastern cooperative oncology group performance score.

A possible objection against the multivariate survival analysis above could be that the follow-up times in the DOD and AWD subgroups partly overlapped. To make possible differences between short-term nonsurvivors and long-term survivors more clear, binary logistic regression analysis was used to study if the features could distinguish between DOD patients in the first survival duration quartile (<11.7 months, *n* = 166), and those who were AWD at the last follow-up in the fourth quartile (≥39.0 months, *n* = 51). Different methods (Enter, Forward, Backward, Stepwise, with *p* < 0.05 to enter and *p* > 0.1 to remove) were used, with approximately the same results (Chi-square 94.3, *p* < 0.0001). Age < vs. ≥75, pathological type, and the number of PBT cycles had independent prognostic value. Once these were included, none of the other features were significant (*p* > 0.10). ROC analysis gave an area under the curve of 0.70 (*p* < 0.001). However, again the sensitivities, specificities, overall correctly classified cases, and positive and negative predictive values were disappointing. The same holds for all other features. The multivariate classifier gave the best results, as **Table 3** and **Figure 3** show.

**Table 3:** Percentages of overall correctly classified cases, sensitivities, specificities, positive, and negative predictive values of features studied to predict alive with disease vs. dead of disease.

		Sensitivity	Specificity	Positive predictive value	Negative predictive value	Overall correctly classified
***Multivariate classifier***		87%	27%	80%	38%	73%
***TNM***		66%	46%	86%	21%	37%
***Gender***		60%	49%	86%	19%	42%
***Sum of TNM, performance score, and pathology subtypes 3 + 4 vs. 5 + 6***		32%	88%	93%	20%	59%
***Pathology adenocarcinoma vs. all others***		31%	82%	90%	19%	61%
***Smoking habits index***		30%	73%	85%	17%	63%
***Performance score***		20%	96%	97%	19%	68%
***Age <75 vs. ≥75***		7%	86%	9%	83%	73%
***EGFR mutation (on 218 of 690 cases only)***		7%	77%	61%	14%	82%

%         Color

<10%

10%–25     
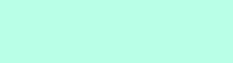

30%–49%     
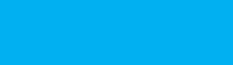

50%–79%     
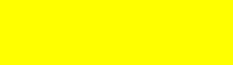

80%–100%    
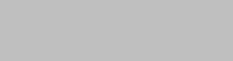

EGFR, epidermal growth factor receptor.

At the start of the study of the long-term survival predictors of patients with late-stage NSCLC, the researchers equipped themselves with the knowledge from studies on stages IIIB–IV patients, that PS, TNM stage, and pathological types would accurately predict prognosis. However, in this stage IV with at least 3 months of survival, this was not the case. The prediction results of the baseline and therapeutic features to distinguish DOD nonsurvivors from long-term AWD survivors is too poor overall to be of clinical value and to answer in individual patients the question “How long do I have?”.

## Discussion

The annual worldwide number of new NSCLC stage IV patients is close to 1 million. In total, 25%–30% die within 3 months, making stage IV NSCLC one of the deadliest cancers. However, little, if any scientific attention has been paid to the question, which factors among the worldwide 700,000 NSCLC stage IV patients, surviving longer than 3 months up to 10 years, cause the good prognosis of the 5%–15% (35,000–105,000) (very) long survivors. It is widely presumed, based on studies in the nearly 1.2 million stages IIIB–IV patients, that age, TNM substage, PS, histopathology, and TKI-TT are significant prognostic factors. However, in the absence of scientific data for the large group of stage IV NSCLC patients surviving at least 3 months, the question from many individuals: “How long do I still have?” currently cannot be reliably answered as scientific data are lacking. We therefore evaluated a large group of 998 stage IV NSCLC patients, of which 261 died within 3 months. In the 737 surviving 3.2–120.0 months, it was evaluated which patients, tumor, and therapeutic factors determine long- and short-term survival.

The first interesting result was the much better than expected 1-, 2-, and 5-year survival rates: 74%, 49%, and 16%. Even at 10 years follow-up, 5% of patients were still AWD. None of these patients surviving 10 years was disease free, which is not surprising as all had stage IV disease at the start of the study. However, the metastases were dormant, and one may expect even longer survival than 10 years (which was the maximum follow-up in the current study). Future studies in the surviving patients therefore would be interesting. Secondly, the 47 patients who refused any form of platinum-based chemotherapy (PBT) or TKI-TT had a worse survival than those who received either of the two or both treatments. They were older (although with considerable overlap), did have a nonsignificant trend towards a worse PS (*p* = 0.07) but substages were not different. The fear for serious side effects of PBT and TKI-TT may have been the major argument of these patients to refuse PBT and TKI-TT. In view of the better survival of PBT and/or TKI-TT patients, it may be considered to explain future stage IV NSCLC patients refusing PBT and TKI-TT, that their prognosis is probably worse if not taking these treatments.

Radiotherapy did not improve prognosis, which was not surprising as it was given in a palliative setting. The number of PBT cycles was strongly prognostic. Patients with 4–6 cycles had a much better survival than those with 1–3 cycles. One could ask why. Patients tolerating standard 4–6 cycles may be in a better physical condition, such as younger or with a (lower performance score) and TNM stage. However, TNM stage and age did not differ (*p* > 0.20), but PS was slightly more often higher (*p* = 0.04) in those with 1–3 cycles. Sixty patients who received TKI-TT but not PBT, had a better prognosis than average (28 rather than 23 months median survival).

With univariate survival analysis, many features were associated with outcome: age, gender, no- or light vs. heavy smoking, PS, TNM substages, pathological type adenocarcinomas vs. others, sum of TNM-PS-pathology, EGFR mutations, the number of PBT cycles and TKI-TT. With multivariate analysis, only baseline features pathological subtype, PS, and TNM IVA/B substages had strong independent survival predictive value, and of the therapy modalities, the number of PBT cycles, and TKI-TT.

We thus could confirm in the stage IV NSCLC patients with more than 3 months survival, the predictive survival significance of the commonly used therapeutic decision criteria. On the other hand, age >75 did NOT have independent multivariate prognostic value. The fact that presence of EGFR mutation also multivariably was not significant can be due to the overshadowing prognostic significance of the pathology adenocarcinoma subtype. Survival analysis showed no survival differences between different locations of the EGFR mutations. However, mutations in 21 were associated with a (just significant) better survival (**Table 1**). Due to the small numbers, we are reluctant to draw strong conclusions. Future large studies are required to confirm this interesting finding.

The survival results of TKI-TT were somewhat worse than expected of previous stages IIIB–IV NSCLC (5, 15, 16). Recently, third-generation epidermal growth factor receptor tyrosine kinase inhibitor (EGFR TKI), osimertinib, demonstrated superior PFS compared with first-generation EGFR TKI in the first-line setting (18.9 months median vs.10.2 months in the EGFR TKI comparator (gefitinib/erlotinib)) (17). Our own 2011 study on stage IV NSCLC also gave stronger prognostic effect for TKI-treated patients, but follow-up time was maximally 30 months (18). We presume that the less favorable results of TKI-TT are due to the fact that the current stage IV NSCLC study is on both adenocarcinomas, squamous, and other pathological cell types, whereas the earlier study only included stage IV adenocarcinomas. Moreover, the follow-up of the current study is much longer. Interestingly, a large nationwide study from Sweden on stages IIIB–IV NSCLC patients showed a significantly better survival for patients with short follow-up (median overall survival (OS): 18.6 months in years 2014–2015) compared with earlier year of diagnosis and hence longer follow-up (15.3 months in years 2010–2011, *p* < 0.05). Note, that the follow-up in the 2014–2015 group in the Swedish study was maximally 36 months (19).

Several of the TKI-TT patients received the treatment without EGFR-mutation analysis, as was usual in the early years of TKI-TT. In retrospect, many of these had squamous or large cell pathology subtypes and had the same poor survival as non-TKI-TT patients. These patients should never have received TKI-TT. When these were left out, the predictive value of TKI-TT strongly improved. Another explanation for the less good than expected survival rates of TKI-TT patients could be that the patients in the current study have very long follow-up, much longer than in all previously published studies. Most patients with advanced NSCLC with EGFR activating mutations will develop resistance after 6–9 months of treatment with first-generation reversible TKIs such as erlotinib, gefitinib, (5, 16, 20). Moreover, earlier TKI studies on adenocarcinomas evaluated all stages IIIB–IV, whereas the current patients all had stage IV NSCLC with >3 months follow-up. This may have been associated with a larger tumor burden than in stages IIIB–IV patients, have led to increased resistance and consequently less good survival.

Despite the strongly significant predictive value of several of the baseline and treatment modalities, none of the features (neither alone nor in combination) can accurately identify subgroups with a short survival (<11.7 months), and relatively long survival (>38 months). The remaining question therefore is, which other candidate features, or interventions could perhaps help to identify and modify short- and long-term survivors among stage IV NSCLC long survivors with >3 months survival.

Attempts to answer this question should keep in mind, that all stage IV NSCLC patients have metastases at the time of diagnosis. Most stage IV NSCLC patients die from metastases when the total growing load becomes so large that it is not compatible with life. In such cases, there are many hallmarks of cancer which can play a role, but 5 main possible features could be especially important to influence the outcome and survival duration:

1. The total number of viable metastatic cells reaches a lethal threshold;
2. The condition of the patient becomes too poor;
3. The response of the body on the tumor cells diminishes under a critical level;
4. The geno- and phenotype and especially proliferation speed of the tumor cells dramatically increase;
5. Interventions influencing these 4 features become insufficient to support life.

Much less-frequent causes of death are life-threatening locations of in itself nonlethal masses, or sudden development of uncontrollable effusions from massive metastases. Unfortunately, we did not have details of the metastatic status of the patients (M1a, M1b, M1c) or localization sites and eventual effusions as death causes. It would be most interesting to study these in a future study.

Very recently, an excellent series of articles was published on the topic emerging biomarkers for NSCLC (11). None of these articles considered NSCLC stage IV only with >3 months survival. Therefore, we will discuss below potentially important biomarkers and possible interventions for this special group.

Important molecular biological features were already implicitly included in the current study, by the choice of TKI-TT type. Yet, others could be important as well. Microsatellite instability (MSI) is a well-known predictive marker for cancer immunotherapy. MSI-high (MSI-H) colorectal cancer is known to be associated with increased tumor-infiltrating lymphocytes (TILs), elevated host systemic immune response, and a favorable prognosis (12). The immune checkpoint molecules CD274, LAG3, and IDO1 expressions in tumor-infiltrating immune cells showed a better prognosis for patients with MSI-H colon cancer. MSI-H causes a build-up of somatic mutations in tumor cells and leads to a spectrum of molecular and biological changes including high tumor mutational burden, increased expression of neoantigens, and abundant tumor-infiltrating lymphocytes (13). Data about the prevalence of MSI among NSCLC are conflicting, and clinical relevance of MSI in TKI is largely unknown. In a series of 480 pulmonary adenocarcinomas, those with a high amount of MSI-H had a higher proliferative activity (39%) than microsatellite stable (MSS) neoplasms (28%) (14). The abundant TILs in MSI-H cancers are especially interesting, as independent studies have shown that TILs play essential roles in the development and progression of different cancer types (21, 22), also in NSCLC (23–25). However, the occurrence of MSI-H in stage IV NSCLC could be very low (14).

Widespread intratumor heterogeneity for both somatic copy-number alterations and mutations has been found in NSCLC. Intratumor heterogeneity mediated through chromosome instability was associated with an increased risk of recurrence or death, a finding that supports the potential value of chromosome instability as a prognostic predictor (26). Driver mutations in EGFR, the MET proto-oncogene, receptor tyrosine kinase protein coding gene (MET), B-raf proto-oncogene (BRAF), and TP53 genes were almost always clonal. Heterogeneous driver alterations later in evolution were found in more than 75% of the tumors and were common in phosphatidylinositol 3-kinase (PIK3CA) and neurofibromin 1 (NF1) genes and in genes that are involved in chromatin modification and DNA damage response and repair. Genome doubling and ongoing dynamic chromosomal instability were associated with intratumor heterogeneity and resulted in parallel evolution of driver somatic copy-number alterations, including amplifications in the cyclin-dependent kinase 4 gene (CDK4), Forkhead box protein A1 gene (FOXA1), and BCL11A genes. Elevated copy-number heterogeneity was associated with an increased risk of recurrence or death (hazard ratio, 4.9, *p* = 4.4 × 10^−4^), which remained significant in multivariate analysis. In 224 patients with EGFR-mutant lung adenocarcinoma treated with EGFR-TKIs, the tumor burden, expressed as the number of metastatic sites at EGFR-TKI treatment, and rapid tumor progression at progressive disease (PD) were predictive of inferior survival in patients with lung adenocarcinoma with activating EGFR mutations (27). Tumor mutational burden (TMB) can also be measured by whole-exome sequencing (WES) or a cancer gene panel (CGP), and these are associated with immunotherapy responses. The recently established CGP named NCC-GP150 with an optimized gene panel size and algorithm is feasible for TMB estimation. This may serve as a potential biomarker of clinical benefit in patients with NSCLC treated with antiprogrammed cell death protein 1 (PD-1) and antiprogrammed cell death-ligand 1 (PD-L1) agents (28). It is unknown if TMB as measured by WES or a CGP, is associated with TKI treatment response. It is also not known if TMB in stage IV NSCLC, with at least 3 months survival, has stronger prognostic value than TNM IVB stage alone.

The large-scale genetic profiling of tumors can identify potentially actionable molecular variants for which approved anticancer drugs are available. However, when patients with such variants are treated with drugs outside of their approved label, successes and failures of targeted therapy are not systematically collected or shared. A highly interesting new approach to test larger gene panels, the drug rediscovery protocol has recently been suggested. This is an adaptive, precision-oncology trial that aims to identify signals of activity in cohorts of patients, with defined tumor types and molecular variants, who are being treated with anticancer drugs outside of their approved label (29). This certainly could be of value for stage IV NSCLC patients with at least 3 months follow-up.

In a very recent study, loss of IL-34 expression is associated with poor prognosis and negative regulation of the immune system of patients with pulmonary adenocarcinoma (30). However, only 18 patients were stage IV, and in these, IL-34 loss and nonloss occurred equally (7/18 and 11/18 patients). Yet, it would be interesting to study IL-34 loss in a much larger group of stage IV NSCLCs with at least 3 months survival.

It is important to consider that all patients in the NSCLC group studied had metastases at the time of diagnosis. The survival differences of the hypothetical prognostic subgroups in **Figure 1** could therefore be explained by differences in proliferation. Proliferation should preferably be measured both in the primary tumor and its metastases. A noninvasive method would be preferable. Recently, a computer tomography (CT) signature consisting of 12 CT features in stage IV EGFR-mutant NSCLC patients with EGFR-TKI therapy, demonstrated good accuracy for discriminating patients with rapid and slow progression to EGFR-TKI therapy (31). Decision curve analysis revealed that the proposed model significantly improved the clinical benefit compared with the clinicopathologic-based characteristics model (*p* < 0.0001). Although the CT signature was developed in one group of patients, and validated in an independent one, multicenter testing of the predictive value and reproducibility of the CT signature remains mandatory.

Can positron emission tomography (PET) scanning be used to measure proliferation in lung cancer? Fluorodeoxyglucose-F18 (18F-FDG) is an indicator of tumor activity *via* glucose metabolism and the most commonly and widely used PET imaging radiotracer. A recent study reported on a new PET tracer 18F-MPG (*N*-(3-chloro-4-fluorophenyl)-7-(2-(2-(2-(2-^18^F-fluoroethoxy) ethoxy) ethoxy) ethoxy)-6-methoxyquinazolin-4-amine), with high specificity to activating EGFR mutant kinase showed significant correlation between tracer uptake and the EGFR mutation status in both preclinical animal models and in patients with NSCLC. The study aimed to identify patients that are sensitive to EGFR-TKIs and to monitor the efficiency of EGFR-TKI therapy. 18F-MPG uptake positively correlated with median progression-free survival. These results are still preliminary, but when confirmed could become valuable (32).

The relationship between proliferation of cancer cells and glucose loading is historically well known since the pioneering work of Otto Warburg. In 1931, he received the Nobel Prize for his work. Today, the correlation between cancer growth and glucose is used worldwide in scanning. *In vitro* studies of NSCLC cells treated with high glucose dosages showed that the RNA-binding protein insulin-like growth factor 2 mRNA-binding protein 1 (IGF2BP1) was highly expressed in high-glucose-treated NSCLC cells. Knockdown of IGF2BP1 inhibited cancer cell proliferation, migration, and invasion, and also induced cell cycle arrest and apoptosis. Also, IGF2BP1 silencing decreased the netrin-1 level in high-glucose (HG)-treated NSCLC cells. Reintroduction of netrin-1 expression rescued IGF2BP1 deficiency-induced cell proliferation reduction, migration suppression, cell cycle arrest, and apoptosis. These findings suggest that IGF2BP1 silencing inhibits the occurrence of tumor events through downregulating netrin-1 expression, indicating that the IGF2BP1/netrin-1 axis exerts an oncogenic role in high-glucose-treated NSCLC cells (33). A recent prospective long-term breast cancer study found that perioperative high intake of glucose was not only associated with high insulin blood levels but also with an extremely poor prognosis (34). This finding could be further correlated with significant metabolomics changes in the blood shortly after the operation (35). A prospective glucose intervention study, or intervention with medicines which can reduce insulin blood levels in stage IV NSCLC patients, therefore would be interesting.

## Conclusions

With 2 million new lung cancers worldwide annually in 2018 (1–3), about 1.6 million are NSCLC and of these 60%–70% (1 million) are in advanced stage IV at the time of diagnosis; 25%–30% of these die within 3 months of diagnosis. The >70% group or worldwide 700,000 stage IV patients per year who survive 3–120 months is very seriously understudied. By extrapolation from all stages IIIB and IV NSCLCs together, it is regarded as having an extremely poor prognosis, but 2- and 5-year survival rates are nearly 50% and 20%. Even at 10 years, still nearly 5% is alive with disease. This percentage may seem low, but speaks much more, when it is considered that with worldwide 5%, regards yearly 35,000 new stage IV AWD patients at 10 years follow-up.

In this study, we have undertaken detailed statistical analysis and quantitative modelling of the survival of a very large group of stage IV NSCLC patients with 3.2–120 months follow-up. The patients seem to comprise different subgroups with widely different survival rates. Multivariable analysis shows that performance score, TNM substage, pathological type, the number of chemocycles, and targeted therapy each have strong independent survival predictive value. However, the multivariate classifier, comprising some or all of these features, could not accurately predict short- and long-term survivors.

We conclude that the question from stage IV NSCLC patients with >3 months survival “Doctor, how long do I have?” cannot yet be answered reliably. To this end, the predictive value of other features and interventions described in detail in the *Discussion*, should be investigated.

## Data Availability Statement

The original contributions presented in the study are included in the article/supplementary material. Further inquiries can be directed to the corresponding author.

## Ethics Statement

This noninterventional, retrospective observational study on patients with first-time onset stage IV NSCLC was approved by the Institutional Research Board of the Longhua University Hospital (LUH), Xuhui district, Shanghai, China before the study commenced. JB later got permission from the Research Director of the Stavanger University Hospital, Stavanger, Norway, to participate. The patients/participants provided their written informed consent to participate in this study.

## Author Contributions

All authors contributed extensively to the work presented in this paper. HG: acquisition of data, analysis and analysis support, interpretation of data, drafting the article or revising it critically for important intellectual content, and final approval. HL: conception and design of the study, acquisition of data, revising the manuscript critically for important intellectual content, and final approval. LZ: acquisition of data, analysis and analysis support, and interpretation of data. JF: acquisition of data, analysis and analysis support, and interpretation of data. XH: acquisition of data, analysis and analysis support, and interpretation of data. JB: advice for analysis, interpretation of results, drafting the article and revising it critically for important intellectual content, and final approval. All authors contributed to the article and approved the submitted version.

## Funding

This study was funded by Three-Year Action Plan for Traditional Chinese Medicine of Shanghai Municipal Commission of Health and Family Planning. No. ZY3-CCCX-3-3023 (ClinicalTrials.gov: NCT02777788). A personal grant No. 2021-721 to JB was obtained from Medical Practice Dr. Jan Baak Inc., Tananger, Norway to participate in this study and the translation correction costs.

## Conflict of Interest

JB runs a private medical practice in addition to the position at Stavanger University Hospital. A personal grant (No. 2021-721) was obtained from this private practice to cover translation costs.

The remaining authors declare that the research was conducted in the absence of any commercial or financial relationships that could be construed as a potential conflict of interest.

## Publisher’s Note

All claims expressed in this article are solely those of the authors and do not necessarily represent those of their affiliated organizations, or those of the publisher, the editors and the reviewers. Any product that may be evaluated in this article, or claim that may be made by its manufacturer, is not guaranteed or endorsed by the publisher.

## Abbreviations

AWD, alive-with-disease; BRAF, B-raf proto-oncogene; BCL11A, *BCL11A* gene; CDK4, cyclin-dependent kinase 4 gene; CGP, cancer gene panel; CT, computer tomography; DNA, desoxyribose nucleic acid; DOD, dead-of-disease; EGFR, epidermal growth factor receptor; FOXA1, Forkhead box *protein* A1 gene; GP, gemcitabine 1,250 mg/m^2^, was administered on days 1 and 8 and cisplatin75 mg/m^2^ was administered on day 1 of a 4-week cycle; HR, hazard ratio; HG, high glucose; IGF2BP1, insulin-like growth factor 2 mRNA-binding protein 1; LUH, Longhua University Hospital; MET, receptor tyrosine kinase protein coding gene; MSI, microsatellite instability; MSI-H, microsatellite instability-high; MSS, microsatelite stable; NCC-GP150, a cancer gene panel called NCC-GP150; netrin-1 (NTN1), protein coding gene, could function as an oncogene; NF1, neurofibromin 1 gene; NP, vinorelbine 25 mg/m^2^, was administered on days 1 and 8 and cisplatin 75 mg/m^2^ was administered on day 1 of a 4-week cycle; NSCLC, nonsmall cell lung cancer; OS, overall survival; P, probability of no difference; PBT, platinum-based chemotherapy; PD, progressive disease; PD-1, programmed cell death protein 1; PD-L1, programmed cell death-ligand 1; PET, positron emission tomography; PFS, progression-free survival; PIK3CA, phosphatidylinositol 3-kinase gene; PS, *Eastern cooperative oncology group performance score*; RNA, ribonucleic acid; ROC, receiver operating curve; RT, radiotherapy; SI, smoking Index; TILs, tumor-infiltrating lymphocytes; TKI, tyrosine kinase inhibitors; TKI-TT, tyrosine kinase inhibitors targeted therapy; TMB, tumor mutational burden; TNM, eight edition of the TNM classification for lung cancer; TP, paclitaxel 135 mg/m^2^ and cisplatin 75 mg/m^2^ was administered on day 1. The cycle was repeated every 4 weeks; TP53, TP53 gene; WES, whole-exome sequencing; 95% CI, 95% confidence interval.
